# The Effect of the Difference in Intensity and Track of Tropical Cyclone on Significant Wave Height and Wave Direction in the Southeast Indian Ocean

**DOI:** 10.1155/2021/5492048

**Published:** 2021-03-03

**Authors:** Yosafat Donni Haryanto, Nelly Florida Riama, Dendi Rona Purnama, Aurel Dwiyana Sigalingging

**Affiliations:** ^1^Department of Meteorology, School of Meteorology Climatology and Geophysics (STMKG), Jl. Perhubungan 1 No. 5, Pondok Aren, Tangerang Selatan 15221, Indonesia; ^2^Center of Research and Development, Indonesian Agency for Meteorology Climatology and Geophysics (BMKG), Jl. Angkasa Pura 1, Kemayoran 10720, Indonesia

## Abstract

This study aims to analyze the effect of the differences in intensity and track of tropical cyclones upon significant wave heights and direction of ocean waves in the southeast Indian Ocean. We used the tropical cyclone data from Japan Aerospace Exploration Agency (JAXA) starting from December 1997 to November 2017. The significant wave height and wave direction data are reanalysis data from Copernicus Marine Environment Monitoring Service (CMEMS), and the mean sea level pressure, surface wind speed, and wind direction data are reanalysis data from European Center for Medium-Range Weather Forecasts (ECMWF) from December 1997 to November 2017. The results show that the significant wave height increases with the increasing intensity of tropical cyclones. Meanwhile, the direction of the waves is influenced by the presence of tropical cyclones when tropical cyclones enter the categories of 3, 4, and 5. Tropical cyclones that move far from land tend to have higher significant wave height and wider affected areas compared to tropical cyclones that move near the mainland following the coastline

## 1. Introduction

The tropical cyclones in the Indian Ocean and the Western Australia ocean have been formed in an average of 10 times per year [1]. The climatology of tropical cyclones in the Australian region shows that the entire northern part of the Australian coastline is vulnerable to the destructive effects of tropical cyclones. A more complete climatology of tropical cyclone events can be found in McBride and Keenan [2] and Holland [3]. There are two main areas of cyclogenesis, namely, the western region above the eastern Indian Ocean and the Timor Sea close to the Indonesian territory and the eastern region above the Coral Sea. Tropical cyclones near the west coast of Australia tend to be stronger than cyclones near the east coast of Australia [3]. The onset of most tropical cyclones in the Australian region can be found around intertropical convergence zone (ITCZ) areas. McBride and Keenan estimate that about 85% of cyclones in Australia have their origin near the ITCZ [2]. Because the ITCZ is located near the Indonesian Maritime Continent or into the north of the Australian continent for most of the tropical cyclone season, most tropical cyclones in the southeast Indian Ocean are near the coast of Western Australia. McBride and Keenan found that about half of all cyclones in their study are formed within 300 km of land [2].

Most tropical cyclones are formed at latitudes 10°–20° from the equator (65%) [4]. Tropical cyclones are influenced by the Coriolis force. The Coriolis force vanishes at the equator, and the tropical cyclone does not cross the equator. However, tropical cyclones can have an indirect impact on the surrounding area, such as storm surges. Therefore, information related to high waves caused by tropical cyclones is very important considering the importance of the ocean as one of the vital transportation routes.

According to Zakir et al. [5], tropical regions that receive solar radiation all year round make tropical oceans warm and form a low-pressure system, and it can make a tropical cyclone if the wind speed near the center is 34 knots or more (63 km/h). A vortex can develop into a tropical cyclone if supported by several parameters. Gray divides these parameters into 2 categories [6].

1. Thermal parameters include ocean thermal energy with sea temperatures >26°C to a depth of 60 meters, the difference in equivalent potential temperature between the surface layers with 500 hPa is more than 10°C, and the presence of wet air in the intermediate layer (>70% at the 700–500 hPa layers)
2. Dynamic parameters include strong vorticity in the lower layer, weak vertical wind shear, and Coriolis parameter at latitude >3°

Wirjohamidjojo and Sugarin [7] classified tropical cyclones based on the wind speed around them. They distinguish three classes, namely, tropical depression (22–33 knots), tropical storm (34–63 knots), and tropical cyclone (>64 knots). Meanwhile, the Bureau of Meteorology has a tropical cyclone scale which is divided into 5 categories [8].

The significant wave height (SWH) was first introduced by Sverdrup and Munk as the average of the highest one-third (33%) of waves (measured from trough to crest) [9]. SWH is one of the important parameters for the statistical distribution of ocean waves. The most common waves are lower in height than SWH, but waves close to double this height can be expected to occur.

The direction of the wave is the direction from which the wave approaches [10]. Waves typically propagate from the center of a storm. Some waves will move in the same direction as the storm, and these will likely grow bigger. Others will head off in the opposite direction, and these will likely lose energy over time and fade away.

The research about tropical cyclone tracks was performed by several researchers in different areas. Ramsay et al. [11] reported clustering tropical cyclone tracks in the southern hemisphere and divided them into 7 clusters. Meanwhile, in the north Indian Ocean region, the tropical cyclone tracks were divided into 6 clusters by Paliwal and Patwardhan [12]. In the Northwest Pacific Ocean region, Camargo et al. distinguished 7 clusters of tropical cyclone tracks [13].

The presence of tropical cyclones also influences significant wave height in the surrounding area. Meanwhile, research on the effects of the tropical cyclone Pabuk by Siregar et al. shows that there is a potential for high waves to reach 4.5 meters to the north of Anambas Ocean and 7.0 meters northeast of Natuna Ocean [14]. Tropical cyclones affect maximum wave height also in the coastal waters of south Java [15].

Based on cyclone reports from BoM (Bureau of Meteorology), examples of tropical cyclones that reach category 5 (severe tropical cyclone) are the tropical cyclones Glenda (27–31 March 2006) and Frederic (25 March‒1 April 1999). The tropical cyclone Glenda caused various damages, such as fallen trees, flooding, and storm tide of about 5.8 meters. Meanwhile, the tropical cyclone Frederic was not recorded as cause damage due to its position far from the coastline. Both tropical cyclones have a unique track and are different from each other. With the difference in cyclone track, we want to analyze the effect of the differences in intensity and track of tropical cyclones on the significant wave height and wave direction, especially in the southeast Indian Ocean.

## 2. Materials and Methods

**Figure 1:**
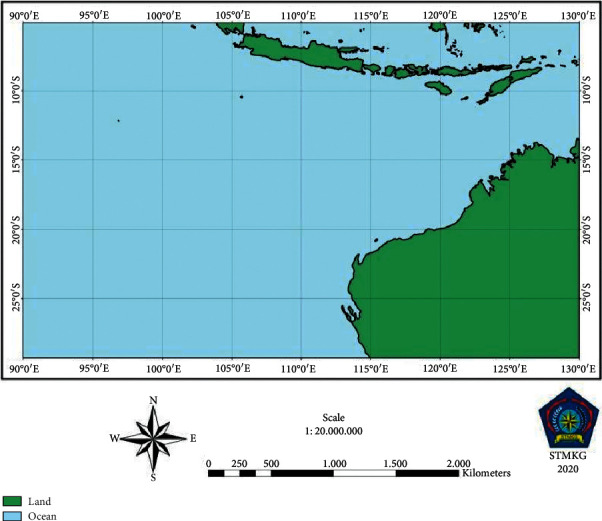
Map of the research area in the southeast Indian Ocean.

The research area is the southeast Indian Ocean, which is limited to coordinates of 5°S–30°S and 90°E–130°E. This region has the most number of tropical cyclones in the world [1]. This area is directly adjacent to the Indonesian Maritime Continent, and in the east, it is bordered by the Australian continent (Figure 1).

Information related to tropical cyclone Frederic on March 25–April 1, 1999, and tropical cyclone Glenda on March 27–31, 2006, existing in the form of a report containing the tropical cyclone position, track, and impacts, were obtained from austrliasevereweather.com. Other than that, the tropical cyclone data were collected from December 1997 to November 2017. This information was used to see the climatological conditions of tropical cyclones in the southeast Indian Ocean. Data were taken in the form of date of occurrence, maximum wind speed, duration, and track of tropical cyclones obtained from Japan Aerospace Exploration Agency (JAXA).

The data of significant wave height and mean wave direction are obtained from the Copernicus Marine Environment Monitoring Service (CMEMS) with a spatial resolution of 0.2 × 0.2° and a temporal resolution of 3 hours. This reanalysis data were taken from December 1997 to November 2017.

Surface wind and mean sea level pressure reanalysis data were obtained from the European Center for Medium-Range Weather Forecasts(ECMWF) ERA-Interim. The temporal resolution is 6 hours, and the spatial resolution is 0.125 × 0.125°.

The method used in this research is a descriptive method in the form of the case study of tropical cyclones. This research begins by identifying the tropical cyclones that were chosen to be studied, namely, the tropical cyclone Frederic and the tropical cyclone Glenda. Furthermore, reanalysis data were collected from CMEMS, ECMWF ERA-Interim, and tropical cyclones data from JAXA for 20 years in the research area.

After all the data have been collected, the next step is to run the data in OpenGrADS application to produce 4 products, namely (1) climatological conditions for significant wave height and wave direction, (2) significant wave height and wave direction condition during the tropical cyclone Frederic and Glenda, (3) surface wind conditions, and (4) mean sea level pressure (MSLP) condition. The results of the OpenGrADS output were analyzed to obtain the conclusions of this study.

## 3. Result and Discussion

### 3.1. Climatology of Number of Tropical Cyclones

**Figure 2:**
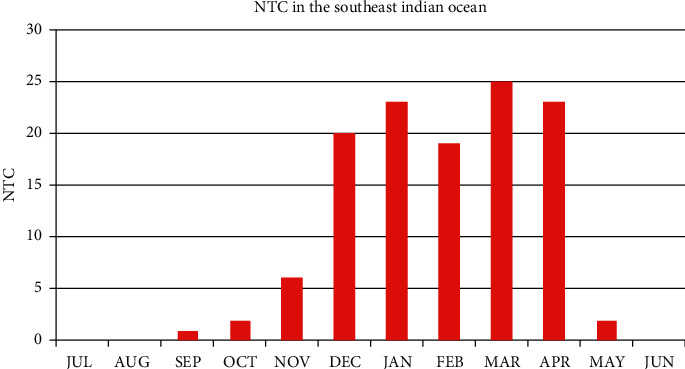
Histogram of seasonal number tropical cyclone events in the southeast Indian Ocean.

This analysis is needed to see the climatology of the number of tropical cyclones in the southeast Indian Ocean.  Figure 2 explains the number of tropical cyclone (NTC) that occurs from December 1997 to November 2017 in the research area.

From Figure 2, the appearance of tropical cyclones in the southeast Indian Ocean from December 1997 to November 2017 occurred most frequently in March with 25 tropical cyclones, followed by January and April in which both had 23 tropical cyclones. Seasonally, the period of December, January, and February (DJF) has the highest number of tropical cyclones with 62 tropical cyclones, followed by the March, April, and May (MAM) period with 50 tropical cyclones. Meanwhile, only a few tropical cyclones occurred during the period of September, October, and November (SON) with a total of 9 tropical cyclones and no tropical cyclones at all in the period of June, July, and August (JJA).

This climatological condition of tropical cyclones is in line with McBride and Keenan's estimates that around 85% of cyclones in Australia have their origin location near ITCZ [2]. The shift of the ITCZ line itself is influenced by the apparent annual motion of the sun, in which during the DJF period, the position of the sun was in the southern hemisphere. The solar radiation received in the southern hemisphere causes the ITCZ to shift southward. Besides, the presence of the sun in the southern hemisphere causes the waters in the southeast Indian Ocean to become warmer and strongly supports the growth of tropical cyclones.

On the other hand, in the JJA period, the position of the sun was in the northern hemisphere and caused the ITCZ to move north. This causes the southeast Indian Ocean to become colder and less supports the growth of tropical cyclones.

The transitional seasons, such as in MAM and SON, still have ocean properties similar to the previous period. After the warm ocean occurred in the DJF period, the MAM period still has the same properties as the DJF, so that the frequency of tropical cyclones in the MAM period is quite a lot. Meanwhile, the ocean properties of the JJA period are still carried over in the SON period and resulted in small frequency of tropical cyclones in these months.

### 3.2. Clusters of Tropical Cyclone Tracks in Southern Hemisphere

**Figure 3:**
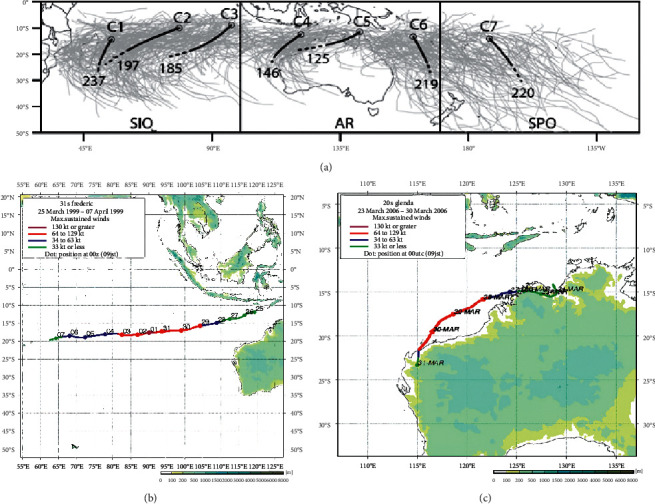
The 7 clusters of tropical cyclone tracks (a) in the southern hemisphere (source: Ramsay et al. [11]) and also the track maps of tropical cyclone (source: JAXA) Frederic (b) and Glenda (c).

The clustering of tropical cyclone tracks in the southern hemisphere has been investigated by Ramsay et al. [11]. In the southeast Indian Ocean, there are 3 clusters of tropical cyclone tracks according to Ramsay et al., namely, C3, C4, and C5 (Figure 3(a)). Each type of cluster has different characteristics from one another.

In this case study, the occurrence of tropical cyclones Frederic and Glenda were taken because they both reached category 5. The track type of tropical cyclone Frederic is C3, while the track type of tropical cyclone Glenda is C4. The tropical cyclone Frederic has a track that is far from the land (Figure 3(b)). Meanwhile, the tropical cyclone Glenda has a track that follows the coastline of northwest Australia (Figure 3(c)). Given the different tracks of these two tropical cyclones, the effect of the difference in intensity and track of tropical cyclone on significant wave height and wave direction will be examined in this study.

### 3.3. Climatology of Significant Wave Height and Wave Direction

**Figure 4:**
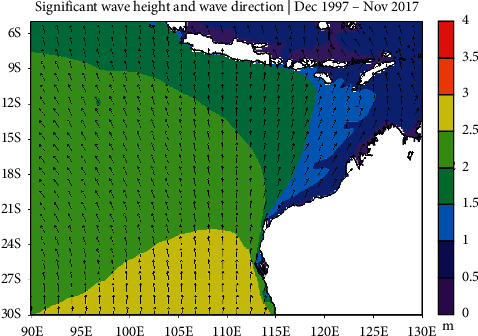
Climatological condition of significant wave height and wave direction for 20 years in the southeast Indian Ocean.

The trend of significant wave heights and wave direction in the southeast Indian Ocean can be understood by looking at the climatological conditions of significant wave heights and wave directions. The climatological condition of significant wave height from December 1997 to November 2017 in the southeast Indian Ocean is shown in Figure 4.

The 20-year average conditions for significant wave height range 0.5–3 meters (Figure 4). Significant wave height in the south of Java Island ranges 1.5–2 meters. Meanwhile, Southern Bali and Nusa Tenggara have significant wave height between 0.5 and 1.5 meters. The climatological condition of significant wave height off the northwest coast of Australia is less than 1.5 meters. As latitude increases, significant wave heights increase to 3 meters in Western Australia. The condition for the mean wave direction for 20 years is almost uniform from the south.

**Figure 5:**
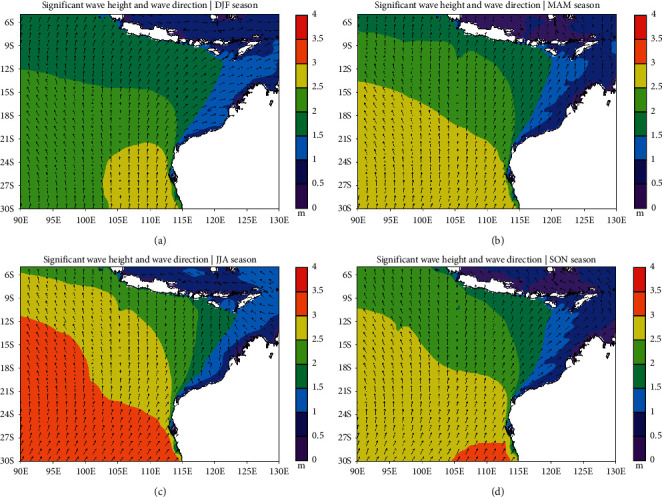
Climatological condition of significant wave height and wave direction for 20 years on the period of DJF (a), MAM (b), JJA (c), and SON (d) in the Southeast Indian Ocean.

The Asian monsoon is active during December, January, and February (DJF). Significant wave height at the DJF period in the southeast Indian Ocean reaches 0.5–3 meters (Figure 5(a)). The condition during the DJF period tends to be lower than the climatological conditions of all periods. The average wave direction condition during the DJF period in the southern regions of the Indonesian Maritime Continent and northwestern Australia tends to come from the south, except in the Java Sea, the wave direction originated from the west-northwest because they followed the direction of the Asian monsoon winds.

During the transition season of MAM, significant wave height condition has increased, especially in the waters of Western Australia (Figure 5(b)). The direction of the waves generally comes from the south. Besides, the direction of the waves in the Java Sea also came from the south, and this was a different condition from the DJF period. This indicates a change in the direction of the monsoon winds.

The Australian monsoon was active during the JJA period, marked by the persistent surface wind direction from Australia to Asia and across the Indonesian Maritime Continent. This greatly affects the increase in significant wave height in the southeast Indian Ocean, which ranges 0.5–3.5 meters (Figure 5(c)). The average of significant wave height in the JJA period is greater than the climatological conditions of all periods. The direction of the waves in the Java Sea originates from the east-southeast, and this corresponds to the Australian monsoon wind conditions. Meanwhile, in most parts of the southeast Indian Ocean, the direction of the waves originated from the south.

The wind starts to become less persistent when entering the SON period. The average of significant wave height in the southeast Indian Ocean during the SON period decreased from the JJA period (Figure 5(d)). The average wave direction condition in the southeast Indian Ocean originates from the south with the direction of the waves in the Java Sea originating from the southeast-south.

### 3.4. Effect of Tropical Cyclone on Significant Wave Height and Wave Direction

In this section, the effect of tropical cyclones on the significant wave height and wave direction at each tropical cyclone's intensity will be discussed. The intensity scale to be discussed is from low pressure to category 5 according to BoM's tropical cyclone scale.

#### 3.4.1. Condition of Low Pressure up to Category 2

Tropical cyclone Frederic gradually strengthened from low pressure to category 2 in MSLP condition, streamline pattern, and significant wave height. As the intensity of a tropical cyclone increases, significant wave height in the surrounding area also increases (not shown). However, the condition of the wave direction around the tropical cyclone has no significant change on a large-scale area.

The tropical cyclone Glenda is formed near the northwest coast of Australia. MSLP condition shows a more drastic decrease when the tropical cyclone increases in intensity until it reaches category 2; even the MSLP value in the tropical cyclone Glenda is lower than the tropical cyclone Frederic (not shown). The streamline pattern also changes with the increase in the intensity of tropical cyclones. The significant wave height does not change when the intensity of Glenda increases to category 2. Similar to Frederic, the wave direction condition at Glenda does not experience significant changes on a large-scale area.

#### 3.4.2. Category 3

**Figure 6:**
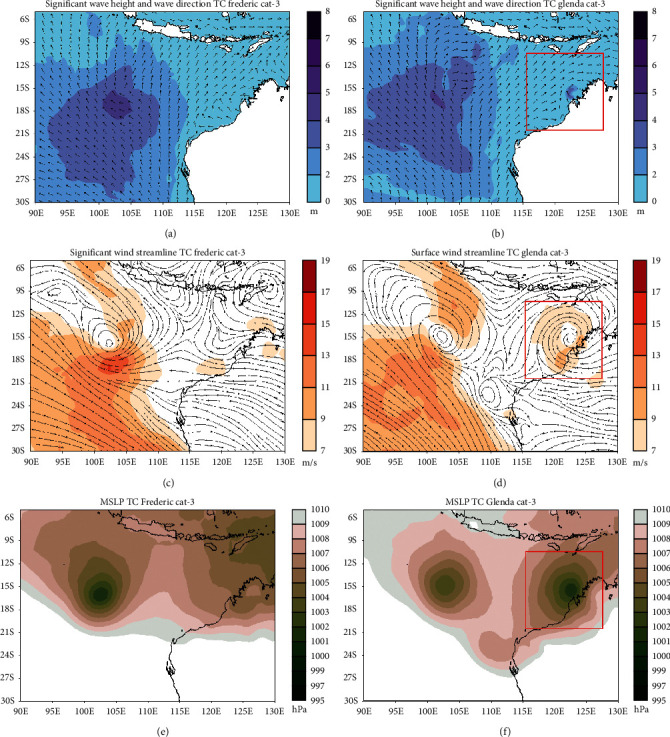
Significant wave height and wave directions conditions during the tropical cyclone Frederic (a) and Glenda (b) phenomena, surface wind speed, and direction at tropical cyclone Frederic (c) and Glenda (d), and mean sea level pressure during tropical cyclone Frederic (e) and Glenda (f). Both tropical cyclones are category 3.

The tropical cyclone Frederic entered category 3 on March 29, 1999, at 06 : 00 UTC. The significant wave height is increasing significantly, especially around the center of Frederic with a range between 4 and 5 meters (Figure 6(a)). In a large-scale area, the wave direction condition changes around the center of Frederic when compared to the climatological conditions of the wave direction in that area. The mean sea level pressure at the center of Frederic ranges 1001-1002 hPa (Figure 6(e)). The cyclonic pattern strengthens as the winds get stronger, with the maximum speed of surface winds around the tropical cyclone Frederic ranging 13–17 m/s in the southern part of the tropical cyclone (Figure 6(c)).

Changes in significant wave height around the center of tropical cyclone Glenda occurred when entering category 3 on March 27, 2006, at 18 : 00 UTC. Significant wave height increases to a range of 2–4 meters (Figure 6(b)). The wave direction pattern changed due to the presence of Glenda category 3. The mean sea level pressure at the center of Glenda ranged 1001-1002 hPa (Figure 6(f)), and this value was higher than the MSLP of Glenda category 2 which ranged 1001-1000 hPa (not shown). The cyclonic pattern on the streamlined map is getting stronger with the maximum surface wind speed around the center of Glenda ranging 9–11 m/s (Figure 6(d)).

#### 3.4.3. Category 4

**Figure 7:**
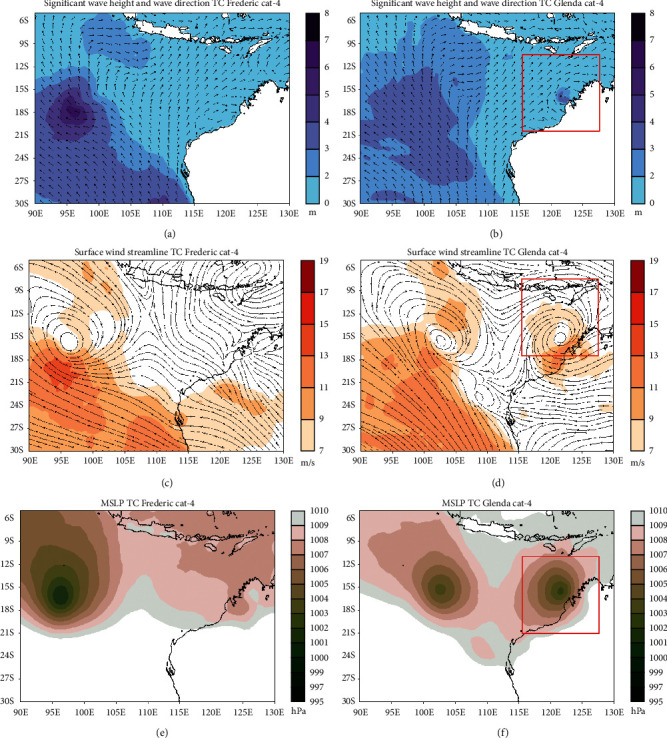
Significant wave height and wave directions conditions during the tropical cyclone Frederic (a) and Glenda (b) phenomena, surface wind speed, and direction at tropical cyclone Frederic (c) and Glenda (d), and mean sea level pressure during tropical cyclone Frederic (e) and Glenda (f). Both tropical cyclone are for category 4.

The presence of tropical cyclone Frederic which entered category 4 on March 30, 1999, at 12 : 00 UTC caused the significant wave height to increase around its center until it reached 7 meters (Figure 7(a)). The wave direction around Frederic originated from southeast-south and moved toward northwestern-north, and this was due to the existence of tropical cyclone Frederic. There was no change in the mean sea level pressure condition in the center of Frederic, which is still in the range of 1001-1002 hPa (Figure 7(e)). The maximum speed of the surface wind ranges 13–15 m/s with a wider maximum wind area (Figure 7(c)). The wind condition appears to be stronger in the south.

In the tropical cyclone Glenda category 4, there is no increase in significant wave height around the center of the tropical cyclone when compared to category 3 (2–4 meters). However, the area affected by the high waves has increased (Figure 7(b)). There was an increase in the mean sea level pressure at the center of Glenda, ranging 1002-1003 hPa (Figure 7(f)). The wave direction experienced a change in a wide area due to the presence of tropical cyclone Glenda category 4 on March 28, 2006, at 06 : 00 UTC. The cyclonic pattern is getting stronger with the maximum speed of surface winds around the center of Glenda ranging from 11‒13 m/s.

#### 3.4.4. Category 5

**Figure 8:**
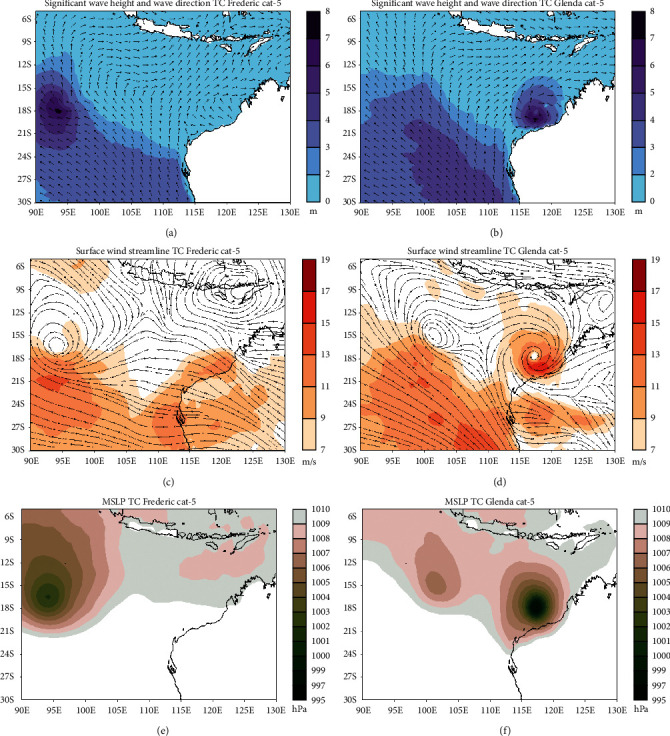
Significant wave height and wave directions conditions during the tropical cyclone Frederic (a) and Glenda (b) phenomena, surface wind speed, and direction at tropical cyclone Frederic (c) and Glenda (d), and mean sea level pressure during tropical cyclone Frederic (e) and Glenda (f). Both tropical cyclone are for category 5.

The tropical cyclone Frederic's status changed to category 5 on March 31, 1999, at 00 : 00 UTC. The significant wave height increases to a range of 6–8 meters near the center of Frederic (Figure 8(a)). There was no change in MSLP at the center of Frederic, which is still in the range of 1001-1002 hPa (Figure 8(e)). The wave direction near Frederic follows the surface wind direction pattern and is widely affected by the presence of tropical cyclone Frederic. The cyclonic pattern strengthens with a maximum speed of surface winds of 13–15 m/s, with areas of strong winds mostly in the south of Frederic (Figure 8(c)).

There was a drastic change in the significant wave height around the tropical cyclone Glenda which was upgraded to category 5 on March 29, 2006, at 12 : 00 UTC, reaching 6–8 meters (Figure 8(b)). The MSLP at the center of a tropical cyclone drops significantly to 995–997 hPa (Figure 8(f)). In Figure 8(d), the maximum speed of the surface winds around the center of Glenda is around 15–17 m/s, and this is much faster than in Frederic.

### 3.5. Discussion

Climatologically, the results of the study show that seasonally tropical cyclones in the southeast Indian Ocean were more prevalent during the DJF period. In these months, the position of the sun is in the southern hemisphere which is summertime. This situation makes the waters of the southeast Indian Ocean warm and strongly supports the growth of tropical cyclones.

From the results of the OpenGrADS output, it is seen that seasonally significant wave height in the southeast Indian Ocean tends to increase with the activity of the Australian monsoon. This causes the JJA period to have the highest significant wave height compared to other periods. Meanwhile, the DJF period has the lowest significant wave height compared to other periods. The direction for all periods is almost uniform from the south.

The significant wave height at the tropical cyclones Frederic and Glenda increases when both tropical cyclones enter categories 3 and 4. Tropical cyclone Frederic has a higher significant wave height and wider affected area compared to the tropical cyclone Glenda. The wave direction around these two tropical cyclones appears to be affected by the presence of tropical cyclones. Surface wind conditions tend to be firmer around the tropical cyclone Frederic compared to the area around the tropical cyclone Glenda. In both, wind speeds tend to be firmer in the south. Faster winds in the southern part of the tropical cyclones have an impact on significant wave heights that tend to be higher in the south of the tropical cyclones.

The height of the significant wave height in both cyclones can reach 8 meters when entering category 5. However, the extent of the affected area is wider in tropical cyclone Frederic. The wave direction near both cyclones follows the direction of the surface wind. The presence of strong winds around Glenda is more even than in Frederic where the strong winds are concentrated in the south of the cyclone.

From the start of low pressure to category 4, there are differences in significant wave heights produced by the two tropical cyclones despite having the same intensity. Tropical cyclone Glenda that are closer to the coastline tends to have lower significant wave height than tropical cyclone Frederic that are far from land. However, when both cyclones reach category 5, significant wave heights are relatively similar and differ only in wide areas, and even then, the position of the tropical cyclone Glenda was far from the shoreline (Figure 8(b)).

The areas with the strongest winds are always in the south, both on the tropical cyclone Frederic (from low pressure to category 5) and on the tropical cyclone Glenda (from categories 3 to 5). This causes the south side of tropical cyclones to have a higher significant wave height than its northern side.

Regarding MSLP at the center of tropical cyclones, the value of MSLP at the center Frederic is more stable and decreased regularly compared to the center of Glenda which is experiencing fluctuations. There is a clear difference when the two cyclones reach category 5 where the tropical cyclone Glenda has a lower MSLP compared to the tropical cyclone Frederic. Tropical cyclone Glenda has a very drastic pressure drop from the previous category.

## 4. Conclusion

Based on the results and discussion carried out in the previous chapter, the following conclusions can be obtained:

1. Tropical cyclones in the southeast Indian Ocean occur most frequently in the DJF months (December, January, and February) during the summer season in the southern hemisphere
2. Significant wave height in the southeast Indian Ocean tends to increase with the activity of the Australian monsoon in the JJA period (June, July, and August)
3. Significant wave height increases with the increasing intensity of tropical cyclones
4. The wave direction is influenced by the presence of tropical cyclones when tropical cyclones enter categories 3, 4, and 5
5. Tropical cyclones that move far from land tend to have higher significant wave height and wider affected areas compared to tropical cyclones that move near land following the coastline
6. Although many tropical cyclones often occur in the DJF period, this does not change the climatological conditions of significant wave height in the DJF months which tend to be the lowest than in other seasons

## Data Availability

The data used to support the findings of this study are provided within this article and are available from the corresponding author upon request.
